# Aerodynamic Noise Simulation of a Super-High-Rise Building Facade with Shark-Like Grooved Skin

**DOI:** 10.3390/biomimetics9090570

**Published:** 2024-09-19

**Authors:** Xueqiang Wang, Guangcai Wen, Yangyang Wei

**Affiliations:** Architecture and Design College, Nanchang University, Nanchang 330031, China

**Keywords:** bionic, super-high-rise buildings, building facade, aerodynamic noise, CFD

## Abstract

The wind-driven aerodynamic noise of super-high-rise building facades not only affects the experience of use inside the building but also reduces the life cycle of building facade materials to some extent. In this paper, we are inspired by the micro-groove structure of shark skin with damping and noise reduction properties and apply bionic skin to reduce the aerodynamic noise impact of super-high-rise buildings. The aerodynamic noise performance of smooth and super-high-rise building models with bionic grooves is simulated via CFD to investigate the noise reduction performance of different bionic groove patterns, such as I-shape, ∪-shape, V-shape, and ∩-shape patterns, and their corresponding acoustic noise reduction mechanisms. This study showed that the bionic shark groove skin has a certain noise reduction effect, and the I-shaped groove has the best noise reduction effect. By applying bionic skin, the aerodynamic noise of super-high-rise buildings can be effectively reduced to improve the use experience and environmental quality of the buildings and provide a new research idea and application direction for the aerodynamic noise reduction design of building facades.

## 1. Introduction

### 1.1. Aerodynamic Noise Issues and Research in Super-High-Rise Buildings

**Figure 1:**
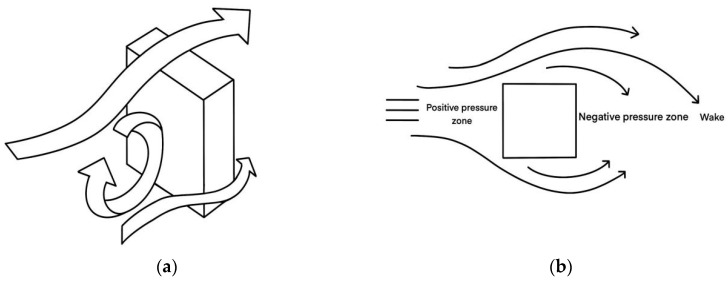
Graphical representation of wind field characteristics in high-rise buildings. (**a**) Schematic diagram of airflow axonometry. (**b**) Schematic diagram of positive and negative airflow pressure.

The global urbanization process has led to an increase in urban population density. To efficiently utilize urban land resources, a large number of super-tall buildings have emerged as an inevitable outcome of high-density urban development. According to the “General Design Code for Civil Buildings” (GB50352-2019) [1], buildings with a height exceeding 100 m are classified as super-tall, regardless of whether they are residential or public buildings. Data from the Council on Tall Buildings and Urban Habitat (CTBUH) indicate that more than 50% of the world’s super-tall buildings exceeding 200 m are located in China [2]. Among them, those between 200 and 300 m dominate, accounting for over 80% of the total super-tall buildings in China, demonstrating the rapid development trend in this field, positioning China as one of the leading countries in super-tall building construction [3]. As building heights increase, so do wind speeds. The high-altitude winds interacting with the surfaces of super-tall buildings create pressure differentials, resulting in aerodynamic noise [4], as show in Figure 1. According to research by Liu Bo [5], in extreme wind conditions, super-tall buildings at heights of 180 m can experience wind noise levels exceeding 90 dB on the building’s surface, surpassing the acoustics standard, which specifies that outdoor environmental noise levels should be below 70 dB [6]. This indicates that aerodynamic noise can significantly affect human auditory comfort under certain conditions, raising concerns about its impact on human health and environmental pollution. Addressing these issues is crucial to identifying methods for reducing aerodynamic noise in super-tall buildings.

Due to the broad sources, uncertainty of noise locations, and irregular variations in outdoor noise for super-high-rise buildings [7], precise in situ measurement of aerodynamic noise poses significant challenges. Additionally, the academic community generally requires stringent and complex measurement protocols for near and far sound fields at heights exceeding 100 m, which are difficult to implement [8]. Furthermore, natural wind tunnel experiments demand advanced hardware facilities, leading to high economic costs [9]. Based on comprehensive studies of aerodynamic noise [10], the academic community predominantly uses Computational Fluid Dynamics (CFD) software for digital modeling and simulations. CFD allows for the rapid and accurate recreation of real wind environments and facilitates the analysis of complex simulation conditions and actual fluid flow data. Commonly used CFD simulation includes STAR-CCM+ and Ansys Fluent. For instance, Tian Hanwen [4] used CFD to simulate wind-induced aerodynamic noise in buildings, revealing that rectangular shapes generate the highest noise levels among different building shapes. Zhang Jintao et al. [11] performed simulations using Ansys Fluent and concluded that noise levels generally increase with building height under the same wind direction. Yuan Tao et al. [12] found that aerodynamic noise levels are highest on the windward side of buildings and that interactions between airflow and building corners significantly contribute to noise. These studies highlight the influence of building shape and height on wind-induced aerodynamic noise, providing the basis for this research, which focuses on CFD simulations of a typical rectangular office complex.

### 1.2. Theoretical and Technological Research on Shark-Like Grooved Epidermis

**Figure 2:**
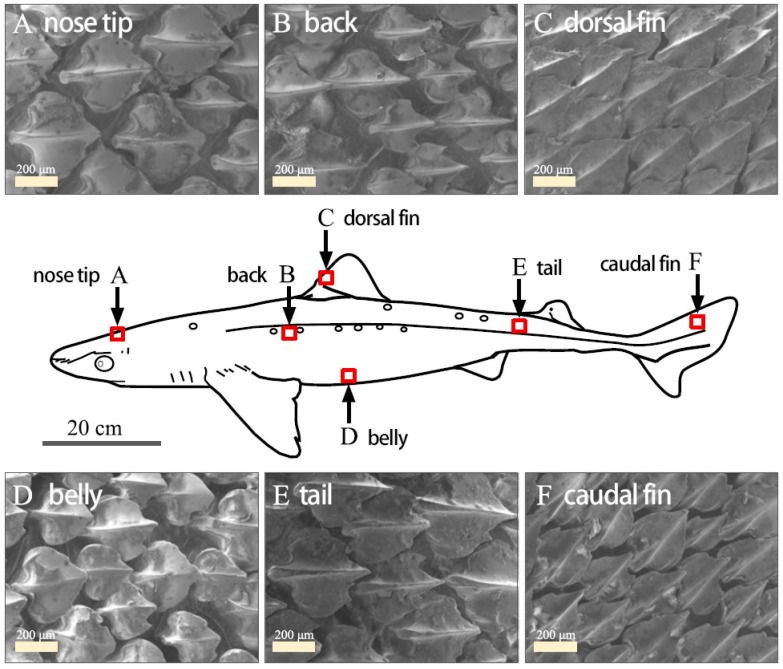
Scanning electron microscopy showing sections of skin at various key positions along the body for a shark.

**Figure 3:**
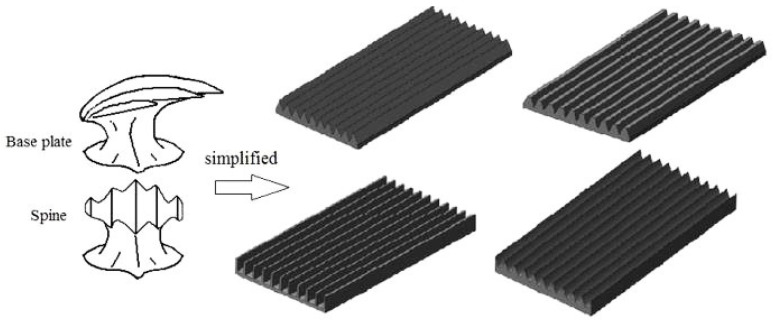
Simplification of real shark skin shield scales into linear micro-grooves.

Shark skin has evolved over millions of years to develop multiple functions such as drag reduction, antifouling, and protection [13]. As shown in the scanning electron microscope (SEM) image of shark scales [14], as show in Figure 2. Researchers discovered that the surface of shark skin is covered with grooved placoid scales [15], which are aligned parallel to the shark’s direction of movement. This alignment effectively reduces wall friction, allowing fluid to flow efficiently over the surface [16]. A series of studies have been conducted on the drag reduction and noise reduction effects of bionic shark skin grooves. Walsh et al. [17] were the first to study the drag reduction of micro-groove structures, simplifying the microstructure of shark skin into grooves of different shapes and sizes for experimental testing [18], as show in Figure 3. The results showed that micro-grooves aligned with the flow direction could effectively reduce wall friction, and the drag reduction effect was found to be related to the dimensionless numbers ℎ+ and s+. Chen Shihong [19] combined CFD simulation and found that a diamond-shaped surface microstructure reduced noise levels by 16.9 decibels compared to a smooth surface on a high-speed train. Hou Zhenhua [20] added bionic shark skin triangular microstructures to the inner surface of exhaust pipes, significantly reducing aerodynamic noise compared to the original exhaust pipe.

At present, there are representative bionic trench drag reduction theories, namely, the prominent height theory and the second vortex group theory, and the analysis of these theories can provide inspiration and a reference for trench noise reduction mechanisms.

**Figure 4:**
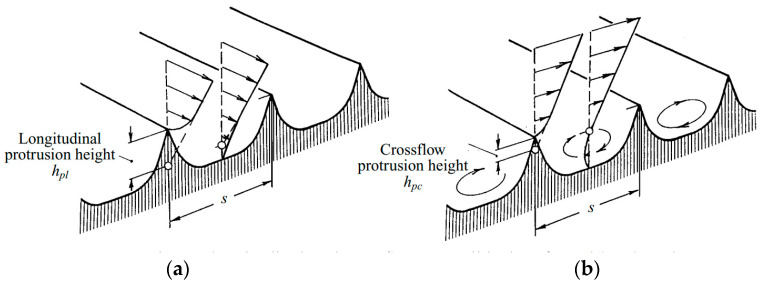
Schematic diagram of the mechanism of surface flow and lateral fluid flow in the groove [22]. (**a**) Flow in the direction of flow. (**b**) Lateral flow and average velocity profile/effective flow starting point.

The “salient height” theory was proposed by Bechert [21]. The “prominence height” theory was proposed by Bechert to explain the drag reduction mechanism of grooves from the point of view of the velocity gradient. The amount to which the height of the groove ridge is displaced vertically above the effective flow start point is called the “effective prominence height”, and the groove raises the effective start point position in the flow, as shown in Figure 4, which is the effective prominence height of the flow. $h_{\mathrm{pL}}$ is the effective protruding height in the flow direction, $h_{\mathrm{pc}}$ is the effective protruding height in the lateral direction, and Δh ($\Delta h=h_{\mathrm{pL}}-h_{\mathrm{pc}}$) is a measure that expresses the reduction in transverse vortex migration at low Reynolds numbers [22]. The bionic groove provides a specific explanation for drag reduction: the bottom of the groove fluid is affected by the viscous effect so that the height of the groove ridge below the fluid velocity gradient decreases. This effect is equivalent to increasing the thickness of the viscous bottom layer so that the average velocity gradient of the wall decreases, which in turn reduces the shear stress of the flow in the boundary layer and achieves drag reduction [23]. This effect is equivalent to increasing the thickness of the viscous bottom layer so that the average velocity gradient at the wall decreases, which in turn reduces the shear stress in the boundary layer and achieves drag reduction.

**Figure 5:**
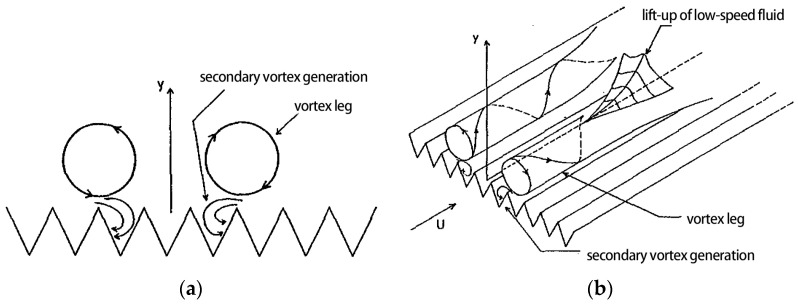
Schematic diagram of the mechanism of the second vortex group theory of grooves [23]. (**a**) Schematic of the flow vortex pair interacting with the groove transversely upward. (**b**) Schematic of the longitudinal groove surface interacting with the flow vortex.

As shown in Figure 5, according to the “second vortex group” theory of Bachert and Smith [23] and Bachert and Smith’s “second vortex group” theory, the drag reduction effect of the groove is due to the interaction of the longitudinal groove surface and downstream “reverse rotation vortex pairs” to form a secondary vortex and multiple grooves under the action of the formation of the secondary vortex group. This secondary vortex group separates the flow vortices from the bottom of the groove, reduces the frequency and intensity of the flow vortex pairs, suppresses the formation of low-speed strips, reduces the momentum exchange in the boundary layer attachment in the flow field, stabilizes the flow field at the bottom of the groove, and achieves drag reduction. Choi [24] et al. confirmed that the groove inhibits the spreading motion of flow vortices, and the flow burst becomes weaker, which is favorable for reducing the surface friction resistance.

**Figure 6:**
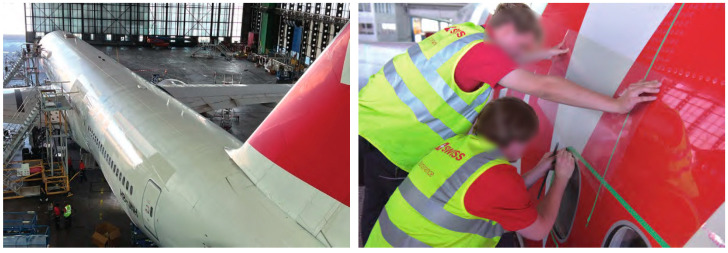
The first “Sharkskin” thin-film Boeing 777-300ER, with technicians applying the “Sharkskin” to the aircraft.

Various bionic shark skin manufacturing technologies are now applied in ships and aircraft. Among these, thin-film drag reduction technology has gained wide application in aerodynamics due to its simplicity, easy maintenance, and cost-effectiveness. For example, Airbus tested fuel-saving thin films with longitudinal micro-grooves on A320 aircraft [25], and Yang Xuefeng [26] achieved large-scale shark skin groove structures using rolling forming technology. On 14 October 2022, the first Boeing 777-300ER equipped with AERO SHARK, a shark skin thin-film surface technology developed by BASF and Lufthansa Technik, was launched [27], as show in Figure 6.

### 1.3. Purpose and Significance of the Study

**Figure 7:**
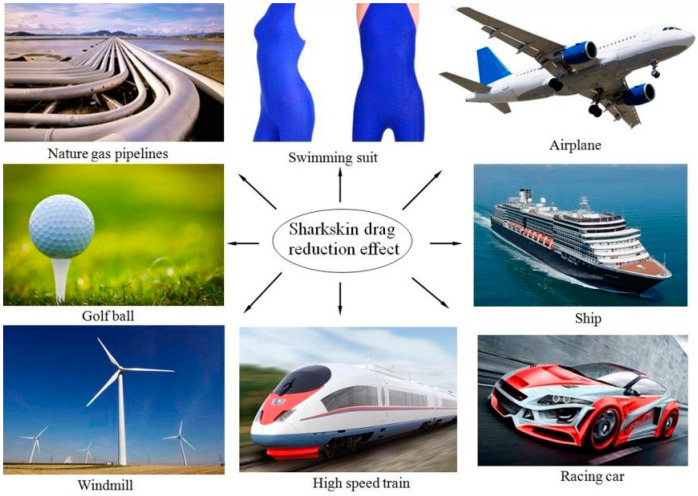
Bionic shark skin surfaces for fluid engineering applications [18].

In the past fifty years, a large number of scientific studies have confirmed that the application of bionic shark grooves has a certain effect on reducing aerodynamic noise, combined with the design of a wide range of high-speed trains, aerospace, automobile manufacturing, and other fields. For example, good results have been achieved for the outer shell of ships, aircraft wing fuselage, and shark skin swimsuits (Figure 7); however, these methods are rarely used in construction engineering. The study of the aerodynamic noise reduction of super-high-rise buildings is based on morphological design. In addition to objectively determining the building form, building facade design is relatively flexible and free, but studies on the aerodynamic noise of facades are very limited, and the lack of architectural designers’ consideration of the reduction in the noise of super-high-rise building facades is obvious [28], especially for special types of aerodynamic noise.

Additionally, bionic shark skin groove films can reduce building heat loss and improve thermal insulation, which lowers energy consumption [29]. These films may also enhance a building’s adaptability by mitigating wind impact on facades and reducing solar radiation and oxidation, thus lowering maintenance costs and extending facade lifespan [30]. Moreover, shark skin structures can reduce dirt accumulation on surfaces [31], decreasing cleaning and maintenance frequency, further reducing energy consumption and costs [32]. Therefore, applying bionic shark groove drag reduction technology on building facades can not only optimize the exterior acoustic environment but also bring multiple benefits in terms of energy and cost savings.

In summary, this study focuses on evaluating the aerodynamic noise reduction performance of bionic films applied to super-tall building facades. By using CFD simulation to model and simulate super-tall building facades, we aim to verify the noise-reducing effect of bionic shark skin grooves under typical wind conditions in high-rise buildings.

## 2. Materials and Methods

### 2.1. Objects of Study

**Figure 8:**
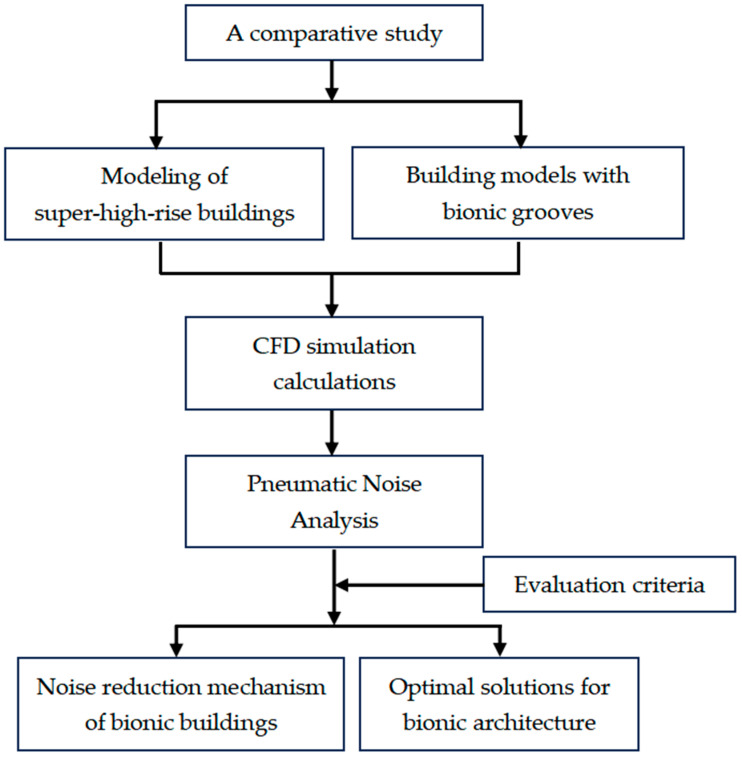
Framework diagram of the overall idea of the study.

Overall, this study established a prototype building of a smooth facade through CFD software and a building model of bionic groove skin, combined with the actual wind field situation of a super-high-rise building in Nanchang, conducted a computer simulation of the sound field environment, carried out a sound field analysis, and derived an optimal model by comparing the noise reduction effect of the bionic structure and combining it with the current noise reduction mechanism. The overall idea of the study is shown in Figure 8.

Super-high-rise buildings are generally designed primarily for commercial office use, with their external appearance categorized into regular and curved forms, and their facades typically enclosed. The rectangular horizontal section is the most prevalent type of floor plan in super-high-rise buildings [4,28]. Moreover, the majority of developed cities in China are located in regions dominated by a subtropical monsoon climate, characterized by prevailing northeast winds in winter and southwest winds in summer. Therefore, the research object of this study is a typical office building with a regular form and a rectangular horizontal section, located in a wind environment dominated by the subtropical monsoon climate, with prevailing winds from either the northeast or the southwest. The building is aligned along a north–south axis based on the rectangular floor plan, making the 45°or 225° diagonal inflow wind directions the primary wind directions considered. A super-high-rise office building in Nanchang, Jiangxi Province, China (latitude and longitude: 115°854679′ E, 28°685879′ N), is selected as an example and its wind environment is simulated. The experiment is conducted with a simplified cube to simulate its building form, and to simplify the simulation computation of the overall experiment, the experiment is conducted with a scaled size of the building model. At the same time, referring to the literature review and calculation formulas, we determine the bionic shark skin groove structure of the facade of a super-high-rise building that conforms to the bionic dimensions and morphology and construct a bionic building model in combination with the prototype building.

#### 2.1.1. Super-High-Rise Building Modeling

The object of this study is a rectangular tower building, which is located in Honggutan District of Nanchang City in the north–central part of Jiangxi Province, China, downstream of the Gan River and Fu River, with a subtropical monsoon climate, a hot summer and cold winter region, an average annual temperature of approximately 17 °C, and an average annual wind speed of 3.48 m/s at a height of 10 m [33]. The annual average wind speed is 3.48 m/s at a height of 10 m throughout the year, and the wind direction is northeastward throughout the year. The height of the building is 303 m, with 59 floors, the plane is a square of 50 m × 50 m, the plane is oriented to the north, south, and below, and the whole building is a typical type of super-high-rise building. The building facade is enclosed and closed in the form of a hidden frame glass curtain wall, and the characteristics of the smooth facade of the research object meet the requirements of this experiment.

At the same time, according to the wind shear theory, in the range of building heights of a typical super-high-rise building, the intensity of the wind speed increases with increasing height [34]. Therefore, the simulation height of the experimental model is positioned at the top 300 m to simulate the maximum impact generated by aerodynamic noise at the top. In this case, the relationship between the wind speed at the height of the building and the height of the building is given by the following equation:

(1) $$v_{1}=v_{0}{\left(H_{0}∕H_{1}\right)}^{\alpha }$$

$v_{1}$ is the wind speed at any position, $v_{0}$ is the standard reference wind speed, $H_{1}$ is the height at any position, $H_{0}$ is the standard reference height, and α is the ground roughness coefficient.

According to Table 1, the research object of this paper is the urban environment, which is the main area of high-rise buildings, for which the power function α is set to 0.3; $v_{0}$ is set to 3.48 m/s; $H_{1}$ is set to 300 m; $H_{0}$ is set to 10 m, from which the calculation of the 300 m height of the annual average wind speed is 9.65 m/s; and the wind direction is northeast, i.e., the experimental gas flow velocity $U_{0}$ is set to 9.65 m/s, with an azimuthal angle of 45° to the building model.

**Figure 9:**
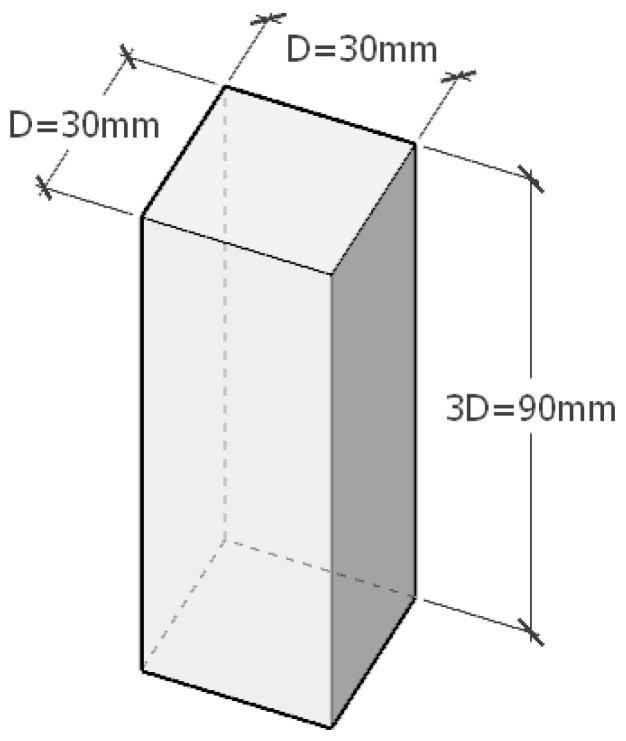
Dimensions of the experimental building model.

Referring to Guo Hao [35] et al.’s building bypassing experiments, the building model in this experiment is set as a super-high-rise building. On the one hand, due to the equal scale modeling of super-high-rise buildings according to the CFD software, the processing size, the calculation area range, and the number of meshes around the form are large, which leads to a considerable computational cost, and at the same time, according to the theory of wind shear, it is beneficial to reasonably reduce the size of the experimental object to ensure the effectiveness and accuracy of the calculations. On the other hand, general sound field experimental modeling requires an appropriate spreading length, which has little effect on the flow field results [36]. Giret [37] et al. conducted sound field experiments with spreading heights of 3.5 D and 7 D, and the calculation results were consistent. Chen Weijie [38] verified that the ideal spreading length of the model is 3 D, which is sufficient for simulating the vortex shedding process of the column bypassing flow, and at the same time, computational resources are not wasted because of the long spreading length. Therefore, in this paper, the spreading length of the numerically simulated smooth rectangular building model is taken as 3 D, i.e., H = 3 D, the side length of the model is D = 30 mm, and the experiments take the D × D × 3 D = 30 mm × 30 mm × 90 mm architectural form as the size of the experimental building model (Figure 9). In addition, the exterior envelope of the experimental object is constructed in the form of a glass curtain wall with a hidden frame, the minor concave-convex construction joints on its surface are negligible, and its smooth facade meets the requirements of the experimental environment.

#### 2.1.2. Bionic Shark Groove Modeling

**Figure 10:**
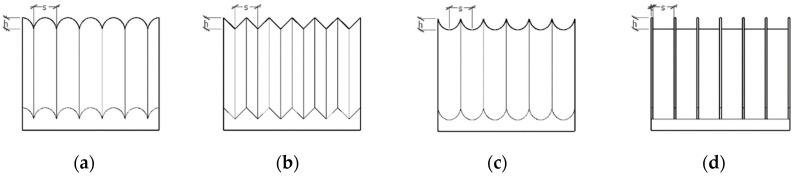
Bionic groove epidermal model: (**a**) ∩-shaped epidermal model. (**b**) V-shaped epidermal model. (**c**) ∪-shaped epidermal model. (**d**) I-shaped epidermal model.

Peet [39,40] et al. used large eddy simulation (LES) to simulate the drag reduction effect of bionic grooves, and the experimental results showed that sinusoidal grooves could reduce drag by 14.5% compared to linear grooves for a certain period arrangement. Bai [41] et al. used the SST-K-W model to calculate the drag reduction effect of six different grooves, and the results showed that knife-edge grooves can achieve a drag reduction effect of 5.6%. Cong Xi [42] et al. used the finite volume method to numerically calculate the flow field on the surface of three kinds of bionic nonsmooth grooves, and their numerical calculation results were consistent with the experimental results of wind tunnels and oil tanks. Among them, the V-shaped groove has the worst drag reduction effect, and the I-shaped groove has the best drag reduction effect, which is mainly because the smaller the top angle of the groove, the larger the slope of the groove valley spacing and the shape curve of the groove surface, and the better the drag reduction effect of the groove surface. Therefore, based on the groove morphology proposed in the above conclusions and the sequential changes in the slopes of the curves of the groove top angle and surface shape, the shark epidermal scales were simplified into four typical groove structures, namely, ∩-shaped, V-shaped, ∪-shaped, and I-shaped (Figure 10).

Walsh [43] et al. proposed dimensionless parameters for trench height h and width s, and the dimensionless dimensions of the height and width of the grooves with a drag reduction effect were $h^{+}$ ≤ 25 and $s^{+}$ ≤ 30, respectively. In addition, Gao Meihong [44] confirmed through further calculations that in the velocity range of 5–100 m/s, the larger the incoming velocity, the smaller the height and width scales of the groove. When the dimensionless height and dimensionless width of the groove satisfy 8.50 ≤ $h^{+}$ ≤ 29.75 and 8.50 ≤ $s^{+}$ ≤ 29.75, the surface of the groove structure only has a drag reduction function when $h^{+}$ = 25.29, the surface of the groove structure has a damping function only when $s^{+}$ = 25.29, and the resistance reduction rate of the groove is maximized. The height and width of the bionic groove are calculated as follows:

(2) $$h^{+}=h\frac{u_{\tau }}{v}$$

(3) $$s^{+}=s\frac{u_{\tau }}{v}$$

where $u_{\tau }$ is the wall shear stress velocity and is the kinematic viscosity.

(4) $$u_{\tau }=\sqrt{\frac{\tau_{w}}{\rho }}$$

$\tau_{w}$ is the wall shear stress, and from the empirical equation for a flat plate, we have

(5) $$\tau_{w}=0.0225\rho U^{2}{\left(\frac{v}{U\delta }\right)}^{\frac{1}{4}}$$

By substituting $\delta =0.37\times {\left(\frac{v}{\mathrm{Ux}}\right)}^{\frac{1}{5}}=0.37\times R_{e}^{-\frac{1}{5}}$ into the above equation, the following equation simplifies to

(6) $$\tau_{w}=0.0296\rho U^{2}Re^{-\frac{1}{5}}$$

Thus, the wall shear stress velocity can be organized as

(7) $$u_{\tau }=0.172URe^{-\frac{1}{10}}$$

Then, the formulas for $h^{+}$ and $s^{+}$ can be organized as follows:

(8) $$h^{+}=\frac{0.172hURe^{-\frac{1}{10}}}{v}$$

(9) $$s^{+}=\frac{0.172sURe^{-\frac{1}{10}}}{v}$$

According to the collation formula, this paper determines the dimensionless size $h^{+}$ = $s^{+}$ = 25 at a $U_{0}$ = 9.65 m/s speed, corresponding to a Reynolds number Re = 6.67 × 10^4^, and determines that the bionic groove structure spacing s = 0.75 mm, height h = 0.75 mm, and width t = 0.2 h = 0.15 mm.

### 2.2. Experimental Procedure

**Figure 11:**
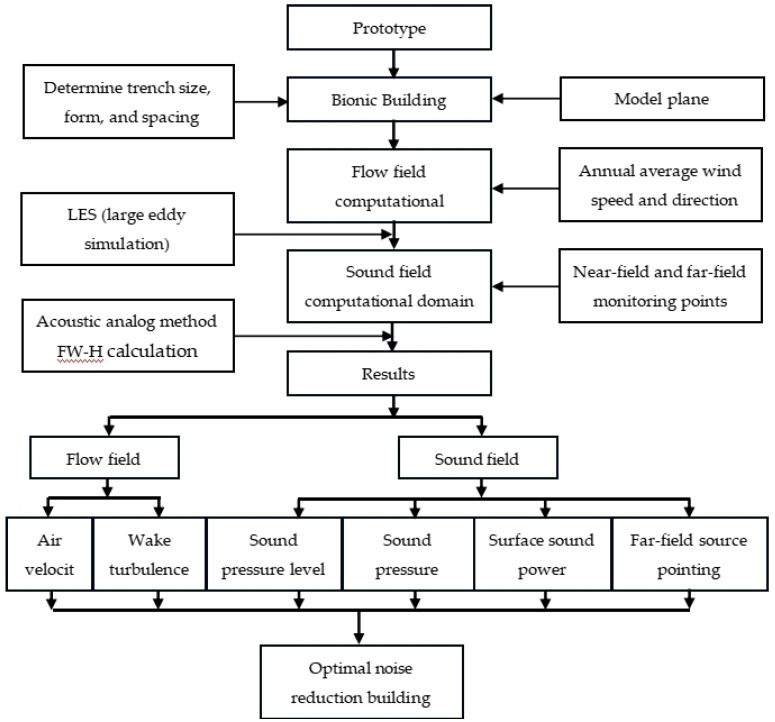
Flow chart of the experiment.

The purpose of the experiment is to assess the aerodynamic noise differences between smooth facade models and facades with various bionic groove structures. The goal is to determine the most effective groove design for noise reduction. Referencing relevant fluid dynamics experiments [45], the experiment follows the procedure outlined in Figure 11. Key geometric parameters such as groove size, shape, and spacing need to be determined during the prototype model preparation phase. The model’s planar orientation will influence wind field simulation and noise propagation. Based on average wind speeds and directions, the flow field domain is set to ensure adequate space surrounding the building model. Reynolds-averaged Navier–Stokes (RANS) equations and the realizable k-ε turbulence model are used to solve the steady-state flow field. Large eddy simulation (LES) is applied to capture detailed turbulence characteristics. Suitable time steps and spatial resolutions are chosen to ensure the accuracy and stability of the simulation. The Ffowcs-Williams–Hawkings (FW-H) acoustic analogy method is used to calculate aerodynamic noise, and parameters such as airflow velocity and wake vortex intensity are analyzed. Noise effects are evaluated by monitoring points in both near-field and far-field conditions. Finally, noise source distribution is visualized through far-field noise source directivity diagrams, helping to identify optimal noise reduction designs.

### 2.3. Modeling

#### 2.3.1. Flow Field Calculation Model

**Figure 12:**
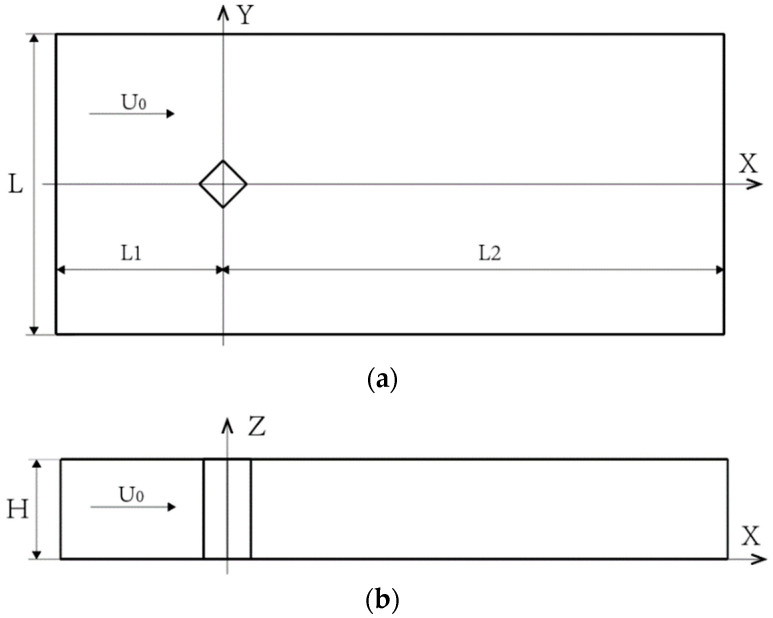
Geometric model of the computational watershed. (**a**) Top view of flow field calculation model. (**b**) Main view of flow field calculation model.

**Figure 13:**
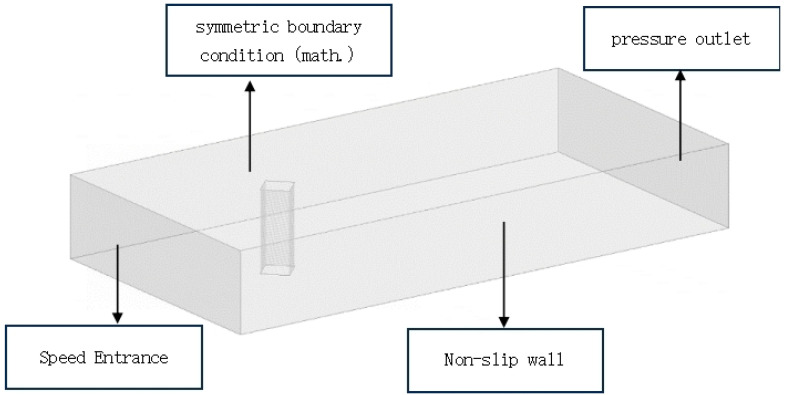
Calculating watershed boundary conditions.

To save a certain amount of computational cost and ensure sufficient computational accuracy, this study needs to determine the appropriate computational area. The computational area is shown in Figure 12: the length of the incoming basin is L1 = 5D, the length of the outgoing basin is L2 = 15D, the width of the basin is L = 10D, and the height of the basin is H = 3D. The boundary conditions are set up as in Figure 13, with the building surface as a non-slip wall boundary condition and the inlet of the computational basin as a velocity inlet. The inlet velocity of the computational basin is set as a velocity inlet. The inlet velocity is $U_{0}$ = 9.65 m/s, the outlet of the flow field is set as the pressure outlet, and the relative pressure is 0 Pa. To eliminate the end effect of the building model, the two end sections of the model spreading direction are set as periodic symmetric surfaces, and the two sides of the outer basin are set as free-slip wall surfaces. On the one hand, to make the flow field converge quickly, the k-ε equation is used for the constant calculation of the flow field, which provides a more stable flow field for the nonconstant calculation; on the other hand, the large eddy simulation (LES) is used for the transient calculation of the flow field based on the constant solution. Air is taken as the fluid medium in the model, the reference pressure is 101.325 kPa, and the air density is 1.29 kg/m^3^.

**Figure 14:**
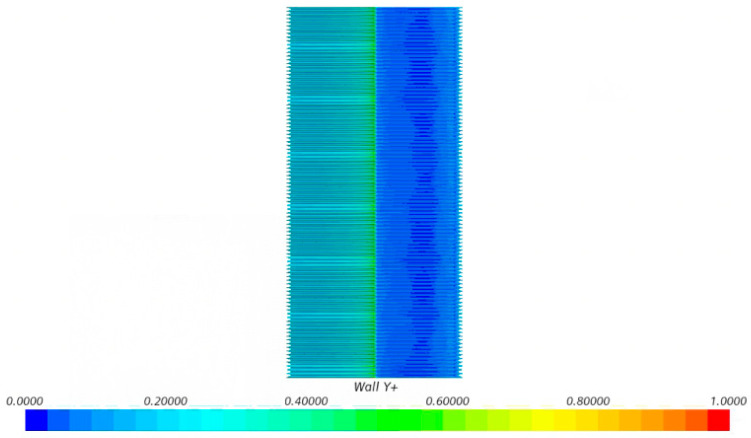
Dimensionless wall distance Y+ plots for bionic skin building models.

**Figure 15:**
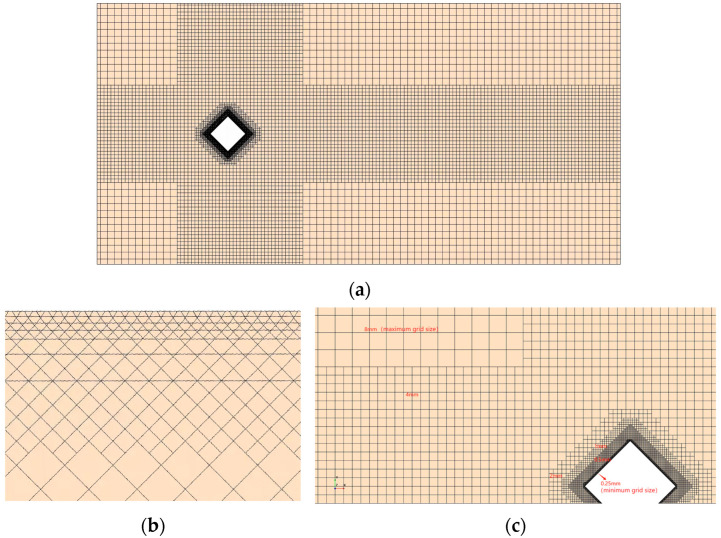
Schematic of the computational watershed grid for the smooth-skinned building model. (**a**) Schematic of the x-y plane grid. (**b**) Enlarged schematic of planar meshing. (**c**) Local view of the model.

**Figure 16:**
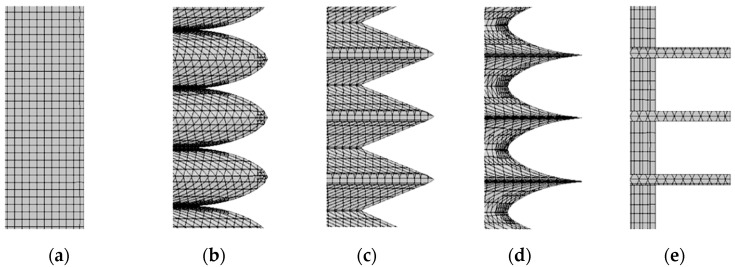
Schematic diagram of the computational watershed grids for the smooth epidermal model and the bionic epidermal model. (**a**) Smooth epidermal model. (**b**) ∩-shaped epidermal model. (**c**) V-shaped epidermal model. (**d**) ∪-shaped epidermal model. (**e**) I-shaped epidermal model.

In terms of model meshing, the five building models were meshed in the same way, and all of them were meshed using Simcenter STAR-CCM+ 2406. At the same time, a more detailed boundary layer was delineated to capture the flow on the surface of the building model, and the height of the first layer of the boundary layer ensured that the dimensionless quantity of the wall, Y+ < 1 (Figure 14). As shown in Figure 15, a structured rectangular grid was used for the computational basin, and a square grid was used for the building model boundary layer to ensure boundary layer delineation. From the local view (Figure 16), it can be seen that the grid nodes are evenly distributed along the circumference of the building, the grid size gradually increases on the boundary layer of the building model, the grid nodes are evenly distributed along the model spread, and the grid size gradually increases from the upper end of the model to the upper surface of the computational basin. The minimum size of the mesh is 0.25 mm, the maximum size is 8 mm, the number of mesh nodes is 10.27 million, and the mesh quality is above 0.8.

#### 2.3.2. Sound Field Calculation Model

**Figure 17:**
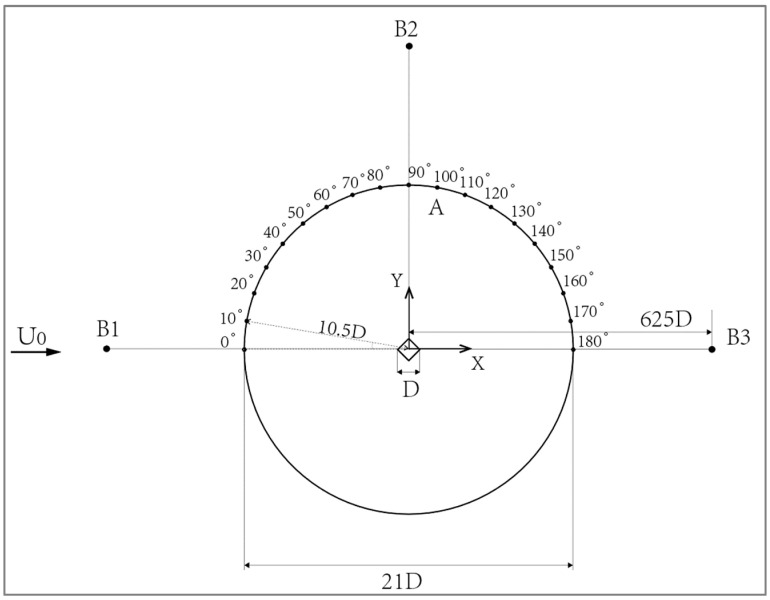
Schematic of monitoring points for sound field calculations.

According to the symmetry plane arrangement of the 2 groups of monitoring points in the near-field and far-field monitoring points, the first group is a number of monitoring points along the building model, and the arrangement is in the center of the horizontal plane from the center of the building at a distance of 10.5D, a diameter of 21D, and a 0–180° uniform arrangement of 19 points, set up as Group A. Group 2 for the three directions from the center of the building floor 625D sets up three monitoring points, B1 (−625D, 0, 0), B2 (0, 625D, 0), and B3 (625D, 0, 0), at this time. The monitoring point from the origin of the distance is much larger than the calculation area, and relative to the monitoring point of Group B, the source can be considered a point source of sound. The arrangement of the monitoring points is shown in Figure 17.

### 2.4. Experimental Process

#### 2.4.1. Simulation Methods and Simulation Process

In this study, the simulation software Simcenter STAR-CCM+ 2406 (Computational Continuum Mechanics) is chosen for the external flow field simulations. This software, developed by CD-adapco, is based on the mechanics of fluid continuous media and is widely used for noise analysis due to its high performance and reliability. The software’s various physical models can meet the Computational Fluid Dynamics (CFD) simulation requirements of this study. Using STAR-CCM+, the study conducted an integrated simulation process that included 3D parameter modeling, grid generation (surface and volume grids), setting physical conditions, solving aerodynamic characteristics of the building model, and post-processing the simulation results, thus efficiently completing the task and shortening the simulation cycle.

Based on the preliminary setup of the square-column building model, flow field and sound field physical environments, grid generation, and boundary conditions, the later simulation process is as follows: First, an appropriate turbulence model such as the realizable k-ε model is selected, and its applicability is discussed. The steady RANS computation is then set up, including parameters like time step size. Steady-state results are used as the initial field for large eddy simulation (LES), where parameters such as subgrid scale are set, and the stability of the flow field is analyzed quantitatively. The surface pulsating pressure is then obtained from the LES results, followed by noise calculations using the FW-H equation. The results of the flow and sound fields, including velocity fields, vorticity fields, sound pressure level spectra, surface sound power, and sound pressure level distribution, are subsequently calculated.

The analysis of the external flow field provides a macroscopic view of the airflow distribution around the building’s facade and the mechanisms generating aerodynamic noise. High-speed airflow may lead to higher aerodynamic noise and greater wind pressure, while the strength and distribution of vortices influence the frequency and amplitude of aerodynamic noise. The external sound field analysis offers a more detailed view at the mesoscopic and microscopic levels of the noise distribution and intensity on the building’s facade. The total sound pressure level assesses the overall aerodynamic noise generated by the building, while the sound pressure spectrum analysis aids in understanding the noise generation mechanism, serving as a basis for designing noise reduction measures. The surface sound power distribution helps us identify the location and intensity of noise sources. Thus, visualized charts and statistical data of the external flow field and sound field are necessary for rigorous noise analysis and comparison.

#### 2.4.2. Computational Flow Field Modeling

For the selection of the flow field model, the flow field is simulated and calculated in this study using the large eddy simulation (LES) turbulence model, which is one of the three simulation methods for calculating the numerical value of the turbulent flow field. LES is one of the three simulation methods used to calculate the turbulent flow field. The large eddy simulation method is a new turbulence numerical simulation method proposed in the 1970s by Joseph Smagorinsky [46]. Joseph Smagorinsky published an article on the “subgrid scale model”. The core idea of the model is to improve the accuracy of the simulation by decomposing the simulated fluid motion and modeling the motion at a scale larger than the computational simulation grid. Large eddy simulation (LES) is an accurate solution for large-scale turbulence motion to capture unsteady turbulence, which overcomes the problem of long computational time for DNS to solve all turbulence scales. LES provides a more reliable means to study the flow mechanism and provides a guide for improving the RANS method, which is widely used in engineering.

The governing equations are as follows: The transient variables are ∅ decomposed into decomposable scale variables $\tilde{\phi }$ and subgrid-scale pulsation variables $\phi^{\prime }$. The control equation is as follows:

(10) $$\emptyset =\tilde{\phi }+\phi^{\prime }$$

where $\tilde{\phi }$ is the variable after filtering and is the part that is directly calculated in the simulation with the LES method; specifically, it can be calculated by the following equation:

(11) $$\tilde{\phi }\left(x\right)=\int_{D}\phi \left(x^{\prime }\right)G\left(x,x^{\prime }\right)dx^{\prime }$$

where D is the flow region,  x ′ is the spatial coordinate in the actual flow region, x is the spatial coordinate in the large-scale space after filtering, and $G\left(x,\;x\;\prime \right)$ is the filter function that determines the vortex scale of the solution.

#### 2.4.3. Solving the Sound Field Model

In the far-field noise solution, this experiment applies the acoustic ratio fitting method based on the area fraction (FW-H), which is a numerical simulation method for computational flow fields. In 1969, Ffowcs-Williams and Hawkings [47] considered the effect of moving solid walls on flow noise and derived the FW-H equation by applying the generalized function method on the basis of the Lighthill–Curle equation. Its control equation is as follows:

(12) $$\frac{1}{c_{0}^{2}}\frac{\partial^{2}P^{\prime }}{\partial t^{2}}-\frac{\partial^{2}P^{\prime }}{\partial x_{i}^{2}}=\frac{\partial^{2}}{\partial x_{i}\partial x_{j}}\left\{T_{ij}H\left(f\right)\right\}-\frac{\partial }{\partial x_{i}}\left[n_{i}p\delta \left(f\right)\Delta f\right]+\frac{\partial }{\partial t}\left[pv_{n}\delta \left(f\right)\Delta f\right]$$

where $\;p\;\prime \left(\;p\;\prime =p-p_{0}\right)$ is the far-field sound pressure, $T_{\mathrm{ij}}$ is the Lighthill stress tensor, $n_{i}$ is the surface normal vector, and $v_{n}$ is the normal velocity.

## 3. Results

### 3.1. Outflow Field Results for the Smooth Skin Building Model and Bionic Groove Skin Building Model

This section takes the external steady state and transient flow field of $U_{0}$ = 9.65 m/s as an example and calculates the flow field around the building model, including the surface airflow velocity cloud, vortex magnitude cloud and horizontal cross-section flow direction vortex cloud. Generally, the vortex movement on the surface of the building model is shown through the velocity distribution and vortex volume distribution, and the flow field situation changes greatly at locations with strong vortex movement.

**Figure 18:**
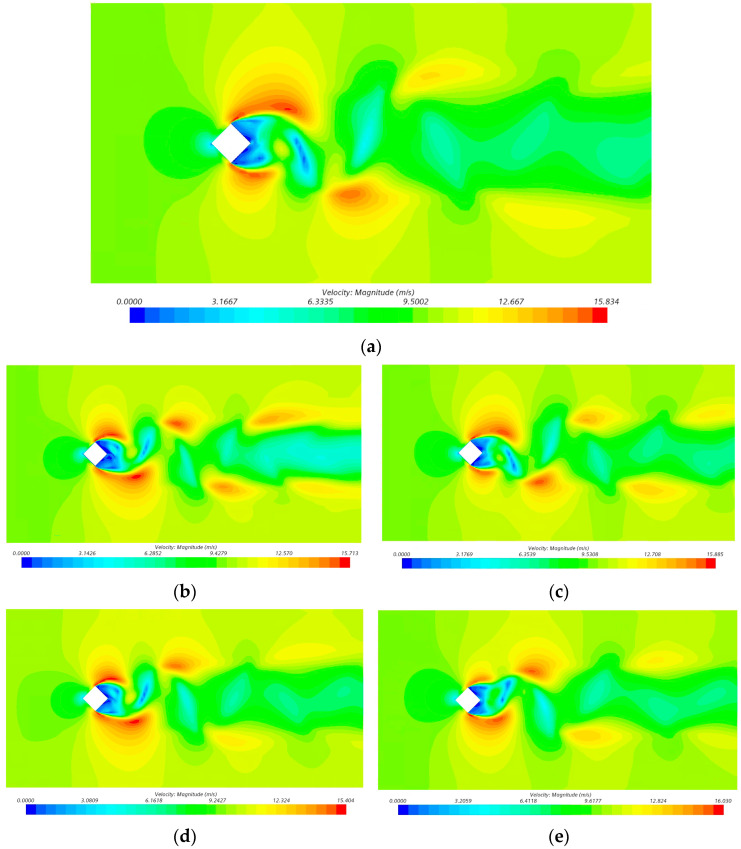
Airflow velocity cloud on the surface of smooth skin building model and bionic skin building model. (**a**) Smooth skin architectural model. (**b**) Architectural modeling of I-shaped groove skins. (**c**) Architectural model of the ∪-shaped groove skin. (**d**) Architectural modeling of the V-shaped groove skin. (**e**) Architectural modeling of the ∩-shaped groove skin.

Figure 18 shows the airflow velocity cloud of the horizontal cross-section of the smooth skin building model and four kinds of bionic groove skin building models. The variation interval of its velocity is 0–16 m/s, the radiation length range is 0–15D, the width range is 0–4D, and the area of drastic airflow change is mainly concentrated on the two sides of the corners and the back of the building model.

**Figure 19:**
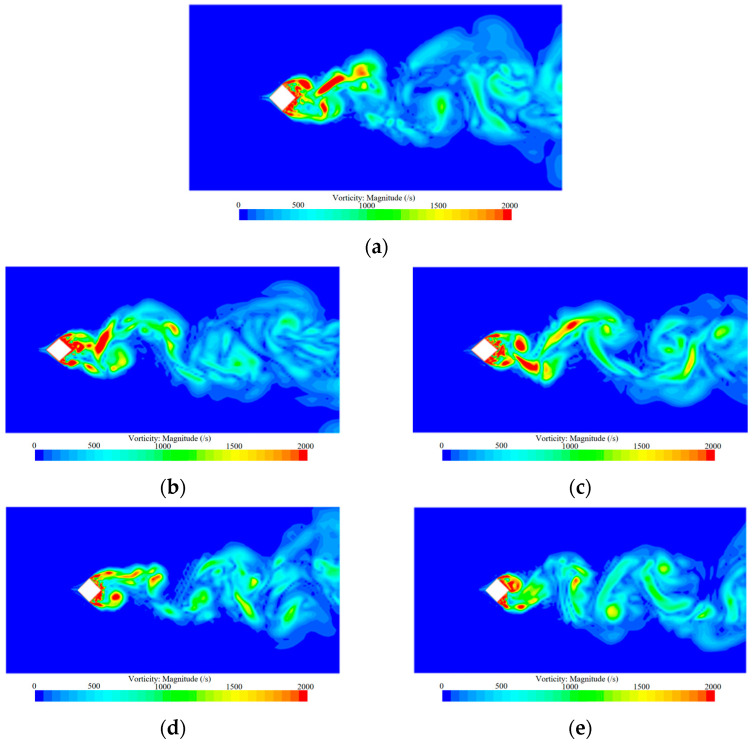
Cloud plot of vortex magnitude for smooth skin building model and bionic skin building model. (**a**) Smooth skin architectural model. (**b**) Architectural model of I-shaped groove skins. (**c**) Architectural model of the ∪-shaped groove skin. (**d**) Architectural model of the V-shaped groove skin. (**e**) Architectural model of the ∩-shaped groove skin.

**Figure 20:**
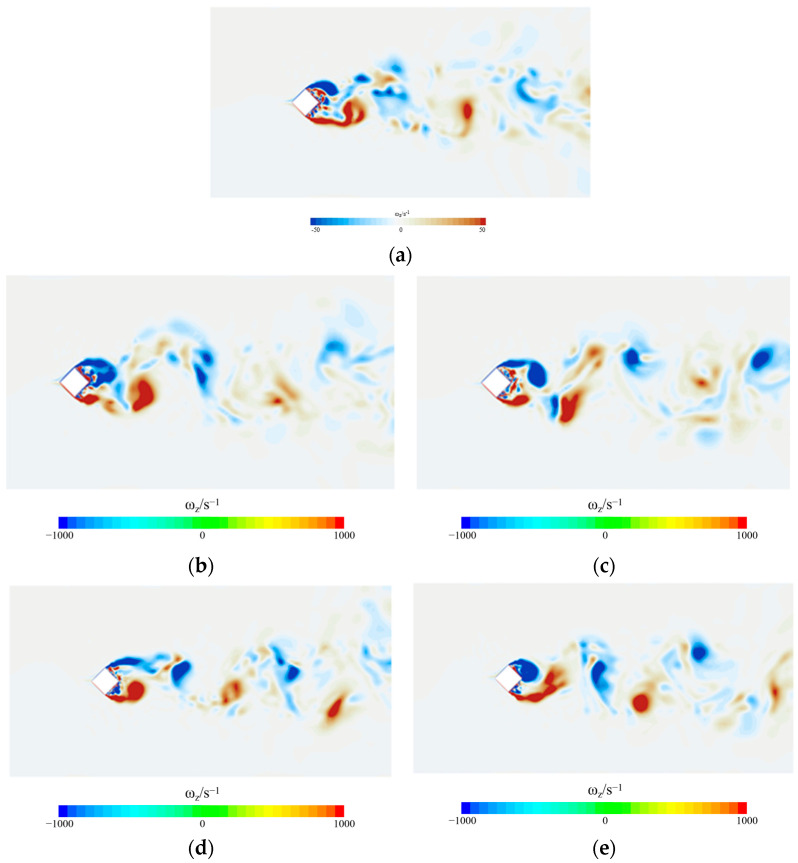
Vortex flow cloud of smooth skin building model and bionic skin trench building model. (**a**) Smooth skin architectural model. (**b**) Architectural model of I-shaped groove skins. (**c**) Architectural model of the ∪-shaped groove skin. (**d**) Architectural model of the V-shaped groove skin. (**e**) Architectural model of the ∩-shaped groove skin.

Figure 19 and Figure 20 show the vortex magnitude and flow direction of the horizontal cross-section of the wake flow when the uniform incoming flow velocity $U_{0}$ = 9.65 m/s in the smooth skin building model and the biomimetic skin building model, respectively, at the calculation time t = 0.450 s. The vortex amplitude and flow direction of the horizontal cross-section of the vortex mainly depend on the change rule and distribution trend of the flow field of the model. In the figure, the up- and downfluctuation amplitudes of the wake flow are obvious, the flow direction is stable, and the change in vortex intensity is mainly concentrated on the side and back of the building model.

### 3.2. Outer Sound Field Results for the Smooth Skin Building Model and Bionic Groove Skin Building Model

The studied external sound field results are presolved with the transient building model for the parameters of the external flow field variables, which are then combined with the turbulence model and flow time to calculate the external flow field noise signal, which is transformed into the total sound pressure level and the associated spectral information via the FFT.

**Figure 21:**
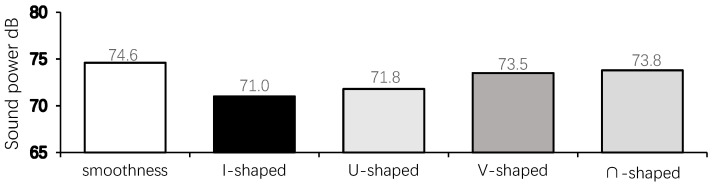
Histogram of maximum sound pressure level of airflow on the surface of the smooth skin building model and the bionic skin trench building model.

Table 2 shows the total sound pressure levels of the smooth skin building model and the bionic skin building model in the far-field B1, B2, and B3 monitoring points. The mean value is the average value of the total sound pressure levels of the three points in Group B, and the mean value is in the range of 18.1–21.7 dB. The amount of noise reduction is the difference between the bionic skin model and the smooth skin model at the monitoring points, the amount of noise reduction of the bionic skin model is in the range of 0.3–2.2 dB, and the percentage of noise reduction is the ratio of the amount of noise reduction of the bionic skin model to the mean value of the sound pressure levels of the smooth skin model, which can be in the range of 1.4–10.5%. The noise reduction percentage is the ratio of the noise reduction of the bionic skin model to the average value of the sound pressure level of the smooth skin model, and the noise reduction percentage of the bionic skin model is in the range of 1.4–10.3%, which can react to the noise reduction effect of the model to a certain extent. Figure 21 is the histogram of the maximum surface sound pressure level of the smooth skin model and the bionic skin model, and the maximum range of the sound pressure level is in the range of 71.0–74.6 dB, which can react to the environmental pollution effect of pneumatic noise. The larger the maximum sound pressure level, the greater the degree of pollution from noise in the environment.

**Figure 22:**
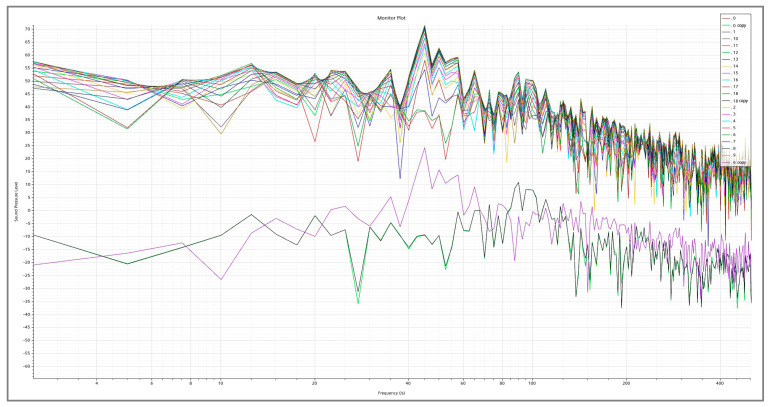
I-shape sound pressure level spectrogram.

Table 3 shows the near-field Group A measurement point sound pressure levels of different building models at every 10° in the range of 0–180°. There is a certain regularity in the directionality of the far-field noise maxima for each trench pattern, with 0° and 180° being the minimum points, and the region where the largest values of far-field noise appear is located in the latter part of the pattern on both sides of the trench perpendicular to the direction of the incoming flow. The measurement point of 100° is the angle with the largest sound pressure level of the five building models, with the highest value in the range of 71.0–74.6 dB. Figure 22 shows the sound pressure level spectrum of the I-shaped groove skin building model with the greatest noise reduction effect, which gradually decreases with increasing frequency, and the flow noise of the building model bypassing the flow is mainly concentrated in the low-frequency band.

**Figure 23:**
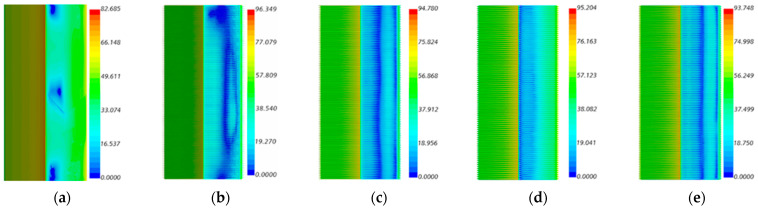
Surface sound power plots of smooth skin building model and bionic skin trench building model. (**a**) Smooth skin architectural model. (**b**) Architectural model of I-shaped groove skins. (**c**) Architectural model of the ∪-shaped groove skin. (**d**) Architectural model of the V-shaped groove skin. (**e**) Architectural model of the ∩-shaped groove skin.

**Figure 24:**
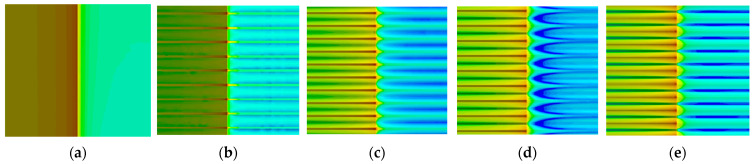
Localized enlarged surface sound power maps in the middle of the smooth skin building model and the bionic skin trench building model. (**a**) Smooth skin architectural model. (**b**) Architectural model of I-shaped groove skins. (**c**) Architectural model of the ∪-shaped groove skin. (**d**) Architectural model of the V-shaped groove skin. (**e**) Architectural model of the ∩-shaped groove skin.

The sound power on the surface of the airflow can determine the distribution of aerodynamic noise on the outer surface of the building model, as shown in Figure 23, for the $U_{0}$ = 9.65 m/s speed of the five models of the side surface of the sound power level distribution cloud. The windward surface distribution is more regular, and the model of the leeward side of the distribution is uneven. Figure 24 shows the model of enlarged turbulence in the corner position, reflecting that the corner on both sides of the sound power changes in the microscopic situation.

## 4. Discussion

### 4.1. Outflow Field Analysis of the Smooth Skin Building Model and Bionic Groove Skin Building Model

As shown in Figure 18, the airflow velocity cloud diagram on the surface of the building model shows that the two sides of the rectangular noise-reducing windward side form a diffuse aerodynamic noise distribution, and the relative flow velocity of the two corners of the model is very fast, while the airflow bypasses the two corners of the model and separates at the back side of the model to form a negative-pressure vortex with a relatively low relative flow velocity. In the velocity cloud diagram of the smooth skin building model, there is a significant change in the velocity of the flow at a distance of 7D in the horizontal basin direction (the instantaneous velocity is more than 12 m/s). Compared with the velocity of the smooth skin building model, the four bionic skin building models exhibit significant changes in the velocity of the wind, the tail flow of the bionic skin building model with an I-shaped groove spreads the furthest, and there is not much change in the velocity of the bionic skin building model with a V-shaped groove and the bionic skin building model with a ∩-shape compared with that of the smooth skin building model. The V-shaped bionic skin building model and the ∩-shaped bionic skin building model do not change much relative to the smooth skin building model. In addition, there is a synchronization between the velocity and vorticity maps, as well as the aerodynamic noise pointing maps, and the location of the wind shadow area corresponds to the presence of greater aerodynamic noise, which shows that the overall wind speed affects the size of the aerodynamic noise, and the local change in the wind speed is not very relevant.

From the vorticity diagram of the wake flow in the horizontal section, it can be seen that when the fluid flows through the architectural model, the separated shear layer is detached from the winding boundary layer, the separated shear layer is detached to form a vortex at the back of the model, and the magnitude of the fluctuations of the vortex in the wake flow does not appear to be significantly reduced. Compared with the wake flow of the smooth skin building model, the vortex flow direction of the wake flow of the bionic skin building model is controlled, the separated shear layer in the wake flow becomes more stable, the process of vortex generation from the coiling of the separated shear layer is slowed, and the magnitude of the up and down fluctuations of the vortex in the wake flow does not show any significant reduction, which indicates that the micro-groove structure of the bionic skin building model can control the transformation of the separated shear layer on the two sides of the model to turbulent flow. At the same time, the frequency of detached shear layer shedding and vortex shedding in the wake flow slows, which reduces the airflow noise caused by vortex shedding. The motion patterns of the external flow field generally conform to the aerodynamic noise generation mechanisms of external building wind fields [4,33].

In summary, under the principle of generating aerodynamic noise, when the facade of a super-high-rise building encounters faster wind or wind around the flow of the building surface, and depending on the location of the corner part of the windward side of the airflow turbulence and vortex generation, the positive and negative pressure differences between the air pressure continue to increase, which stimulates aerodynamic noise.

### 4.2. External Sound Field Analysis of the Smooth Skin Building Model and Bionic Groove Skin Building Model

**Figure 25:**
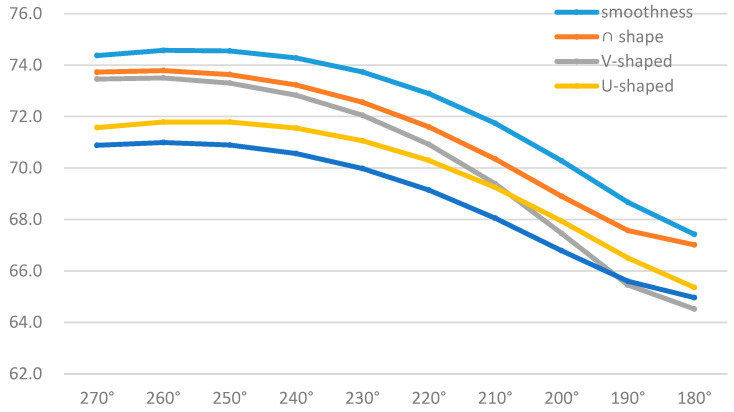
Sound pressure level plots of smooth skin building model vs. bionic skin trench building model in Group A.

From Figure 25 combined with Table 2 and Table 3, it can be concluded that the average near-field aerodynamic noise SPL of each form of groove skin is ranked as follows: smooth skin > ∩ groove skin > V groove skin > U groove skin > I groove skin. The average far-field aerodynamic noise SPL is ranked as smooth skin > ∩ groove skin > V groove skin > U groove skin > I groove skin, which is consistent with the two rankings. The maximum sound pressure level of the smooth skin building model is 74.6 dB, and the I-shaped bionic skin model has the largest noise reduction, which is 10.3%, followed by the U-shaped, V-shaped, and ∩-shaped models. The results align with CONG Qian’s findings regarding the noise reduction effectiveness of bionic groove structures [42], demonstrating the validity of this noise reduction method.

**Figure 26:**
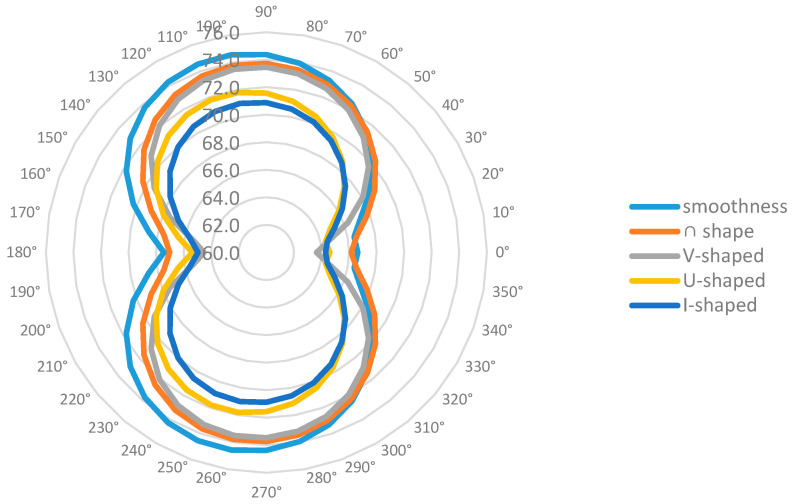
Sound pressure level pointing plot of smooth skin trench building model vs. bionic skin trench building model in Group A.

In the spectrogram of the sound pressure level at the monitoring point of Group B, the flow noise of the model winding is mainly concentrated in the middle- and low-frequency bands, and the sound pressure magnitude in the middle- and high-frequency ranges shows a decreasing trend, so reducing the middle- and low-frequency noise can significantly reduce the aerodynamic noise generated by the model winding. According to the surface sound power diagram, the surface sound power of the smooth skin model is larger and uneven, concentrating on the two top and middle positions on the back of the model, while the surface sound power of the bionic skin model is relatively small and uniformly distributed. The areas with higher sound power are concentrated in the center corner position; the areas with lower sound power are concentrated in the back position. According to the surface airflow maximum sound pressure level diagram, the maximum near-field aerodynamic noise sound pressure level of each shape of the grooved skin decreased in the following order: smooth skin > ∩-shaped grooved skin > V-shaped grooved skin > U-shaped grooved skin > I-shaped grooved skin. The overall amplitude of the sound pressure level of the bionic skin model is larger than that of the smooth skin model, with a maximum difference of 15.9 dB and a maximum sound pressure level of 96.3 dB, which indicates that the bionic skin model has a relatively significant effect on noise reduction. From the sound pointing diagrams of the smooth skin model and the bionic skin model given in Figure 26, it can be seen that the sound source characteristics of the bionic skin model are similar to those of the dipole model, which indicates that the aerodynamic noise caused by bypassing is due to the transient pressure pulsation of the wall caused by the vortex that the airflow over the surface of the model sheds alternately; therefore, reducing the amplitude of the pressure pulsation can effectively reduce the noise of the dipole generated by the model bypassing. In addition, the maximum radiated sound pressure level is concentrated on the axis perpendicular to the inflow direction of the airflow, i.e., approximately 100° of the incidence angle. Therefore, the axis perpendicular to the airflow inflow direction is the dipole axis, and the sound pressure measured in the orientation consistent with the incoming direction of the airflow is very small, indicating that this part of the sound pressure is generated by the quadrupole sound source, i.e., the turbulent stress generated when the vortex model is shed. The sound pressure level spectrum and directivity diagram show distributions consistent with the noise characteristics of cylindrical structures studied by Huang Hui et al. [45], indicating that the noise characteristics of the rectangular-plan building are similar to those of square columns.

The experimental noise reduction ordering of the bionic trench skin is also in line with the theory of the bionic trench drag reduction mechanism [38], i.e., the smaller the top angle of the groove, the greater the slope of the groove valley spacing and the greater the groove surface shape curve, the greater the lifted of the secondary vortex, the greater the thickness of the laminar bottom layer, and the greater the influence of the spreading vortex of the flow-oriented vortex in the turbulent proposed order structure, the greater the ability of the bionic groove to reduce damping and noise.

## 5. Conclusions

Ruben and Julian [48,49] have demonstrated a more systematic application of bionics by utilizing computer tools to integrate extensive biological knowledge into a framework suitable for engineering applications. Similarly, this study applies computer tools to integrate biology and architectural engineering to achieve practical applications of bionics. Using Simcenter STAR-CCM+ 2406 simulation and combining LES with the FW-H method, the aerodynamic noise characteristics of both smooth-surface building models and four types of bionic shark groove facade models were calculated and compared. The analysis of flow and sound field results, along with noise reduction mechanisms, led to the following conclusions:

Under a diagonal inflow wind direction of 9.65 m/s, the rectangular-plan building model with a bionic shark groove surface structure demonstrated a certain degree of noise reduction compared to the smooth-surface building model, with a maximum reduction of up to 10.3%. The ranking of surface noise reduction effectiveness was I-shaped groove > U-shaped groove > V-shaped groove > ∩-shaped groove > smooth surface. For the rectangular-plan building under diagonal inflow conditions, aerodynamic noise was mainly generated at the sharp corners of the windward side, with the minimum value at the center point of the windward side (0°) and the maximum at 100°. Noise intensity was concentrated on the leeward side of the building, and the distribution was influenced by the shape of the bionic grooves. Far-field noise levels were also highest at point B2 (90°) and lowest at point B1 (0°), consistent with the near-field noise distribution.

Based on the research results, if the prevailing wind direction of a city aligns with the diagonal inflow wind direction relative to the rectangular-plan super-high-rise building, subsequent noise reduction measures can be determined. These may include the positioning of transparent bionic surface membranes for glass maintenance and the arrangement of flat or double-glazed glass during early construction. Notably, noise reduction measures may not be necessary for the central or lateral windward facades, but as the form extends toward the leeward region, transparent bionic membranes or double-glazed windows may be required.

The innovative value of this study lies in its focus on reducing aerodynamic noise for super-high-rise rectangular-plan office buildings exceeding 100 m in height, situated in typical subtropical monsoon climates of mainland China, with a specific inflow wind direction at a 45° angle, i.e., a diagonal inflow wind direction. While previous research on drag reduction and noise control has largely focused on streamlined bionic cylinders in the fields of aerodynamics and transportation, studies on square-column buildings remain scarce [32]. This study offers a new direction for addressing aerodynamic noise in the design of super-tall buildings. The results indicate that V-shaped grooves achieve a noise reduction effect comparable to that of I-shaped grooves [40], which are considered optimal for drag reduction in industrial applications. Further comparison of these groove models in practical facade applications is needed. Additionally, the impact of bionic films on other physical aspects of building facades, such as lighting and reflection, requires further investigation, taking into account material limitations, structural technology, cost efficiency, and environmental adaptation. This study only conducted numerical simulations for the wind environment of Nanchang, China, and lacks diverse data sample analysis and tests on different building plan shapes and wind directions in various cities. Additionally, physical experiments in natural wind environments are needed to verify the effectiveness of the findings. Future research should consider different building shapes, various super-high-rise building groupings, and natural wind field variables. These will be key areas for the further refinement and deepening of this research. Continued investigation will aim to address current limitations, thereby promoting more efficient and environmentally friendly noise reduction methods for super-high-rise building facades.

## Figures and Tables

**Table 1:** Range of the highest gradient winds with their corresponding coefficients in various landforms.

Topographic Classification	Height h	The Coefficient α
Offshore sea, islands, coasts, lakeshores and desert areas	300	0.12
Fields, countryside, jungles, hills and sparsely housed townships	350	0.16
Urban areas with dense built-up areas	400	0.22
Urban areas with densely built-up urban areas and taller houses	450	0.3

**Table 2:** Graph of total sound pressure levels at far-field Group B monitoring points.

	B1/dB	B2/dB	B3/dB	Average Value	Noise Reduction	Noise Reduction Percentage
smoothness	18.6	26.6	18.6	21.3	--	--
∩-shape	19.0	26.9	19.1	21.7	0.4	1.9%
V-shaped	18.2	26.5	18.2	21.0	0.3	1.4%
U-shaped	16.4	24.4	16.4	19.1	1.2	5.6%
I-shaped	15.3	23.6	15.3	18.1	2.2	10.3%

**Table 3:** Distribution of total sound pressure levels in near-field Group A (dB).

	0°	10°	20°	30°	40°	50°	60°	70°	80°	90°	100°	110°	120°	130°	140°	150°	160°	170°	180°	Average Value
smoothness	66.6	66.5	67.3	68.6	70.1	71.4	72.4	73.3	73.9	74.4	74.6	74.5	74.3	73.7	72.9	71.7	70.3	68.7	67.4	71.2
∩-shape	66.2	66.6	67.8	69.1	70.3	71.4	72.3	73.0	73.5	73.7	73.8	73.6	73.2	72.6	71.6	70.4	68.9	67.6	67.0	70.7
V-shaped	63.6	64.5	66.3	68.1	69.7	70.9	71.9	72.7	73.2	73.5	73.5	73.3	72.8	72.0	70.9	69.4	67.5	65.5	64.5	69.7
U-shaped	64.6	64.4	64.9	66.1	67.4	68.6	69.7	70.5	71.1	71.6	71.8	71.8	71.6	71.1	70.3	69.2	67.9	66.5	65.4	68.7
I-shaped	64.3	64.5	65.3	66.4	67.5	68.5	69.4	70.1	70.6	70.9	71.0	70.9	70.6	70.0	69.1	68.1	66.8	65.6	65.0	68.1

## Data Availability

Data are available upon request.
